# Femtosecond-laser-irradiation-induced structural organization and crystallinity of Bi_2_WO_6_

**DOI:** 10.1038/s41598-020-61524-y

**Published:** 2020-03-12

**Authors:** Ivo M. Pinatti, Amanda F. Gouveia, C. Doñate-Buendía, Gladys Mínguez-Vega, Juan Andrés, Elson Longo

**Affiliations:** 1 CDMF, LIEC, Federal University of São Carlos (UFSCar), P.O. Box 676, São Carlos, 13565-905 Brazil; 2 GROC, Universitat Jaume I (UJI), Institut de Noves Tecnologies de la Imatge (INIT), Castelló, 12071 Spain; 3 Department of Analytical and Physical Chemistry, University Jaume I (UJI), Castelló, 12071 Spain

**Keywords:** Design, synthesis and processing, Ceramics

## Abstract

Controlling the structural organization and crystallinity of functional oxides is key to enhancing their performance in technological applications. In this work, we report a strong enhancement of the structural organization and crystallinity of Bi_2_WO_6_ samples synthetized by a microwave-assisted hydrothermal method after exposing them to femtosecond laser irradiation. X-ray diffraction, UV-vis and Raman spectroscopies, photoluminescence emissions, energy dispersive spectroscopy, field emission scanning electron microscopy, and transmission electron microscopy were employed to characterize the as-synthetized samples. To complement and rationalize the experimental results, first-principles calculations were employed to study the effects of femtosecond laser irradiation. Structural and electronic effects induced by femtosecond laser irradiation enhance the long-range crystallinity while decreasing the free carrier density, as it takes place in the amorphous and liquid states. These effects can be considered a clear cut case of surface-enhanced Raman scattering.

## Introduction

Bismuth tungstate, Bi_2_WO_6_ (BWO), is an important *n*-type semiconductor with a narrowband gap energy (*E*_gap_) of 2.8 eV that allows efficient absorption of visible light (λ > 400 nm) and has been widely studied due to its wide range of properties, such as ferroelectricity, piezoelectricity, pyroelectricity, nonlinear dielectric susceptibility, and photoluminescent emissions^1,2^. The multifunctionality of this material has been demonstrated on its photocatalytic activity^3–22^, photocatalytic degradation of drugs^23^, dyes^24–26^ alcohols^27^, and phenol^28^, environmental purification, energy conversion^29^, and production of sustainable and combustible compounds^3^. Recently, some works attested this material to be efficient in water splitting for hydrogen generation^30–32^, for shielding against low-energy gamma rays^33^ and for CT/IR imaging and photothermal/photodynamic therapy of tumours^34,35^.

BWO has an orthorhombic structure and belongs to the *Aurivillius* family. It is formed by alternating (Bi_2_O_2_)_n_^2n+^ and perovskite (WO_4_)_n_^2*n*^ layers^36^, which are composed of (WO_6_) octahedral layers and (Bi–O–Bi) layers. Their crystal structure can also be described by alternating [Bi_2_O_2_]^2+^ slabs and [WO_4_]^2−^ slabs, with oxygen atoms shared between the slabs to create chemical bonds. An important characteristic of this structure is that local environments of both W and Bi cations are highly distorted^37^. The off-centre octahedral distortions are a general feature of the structural chemistry of *d*^0^ metal cations. Each W cation is coordinated with six O atoms to form [WO_6_] octahedral clusters that are connected to each other by corner-sharing O atoms. The (Bi_2_O_2_)^2+^ layers are sandwiched between (WO_6_) octahedral layers. From an electronic point of view, BWO presents hybridized valence bands occupied by the Bi 6 *s* and O 2*p* states that shift the absorption band edge towards the visible region, with the concomitant appearance of a narrow absorption region^38–42^. Therefore, an interesting feature of the BWO orthorhombic structure is the innate presence of both crystal distortion and Bi 6 *s* lone pair electrons, which are associated with structural and electronic order/disorder effects, respectively, that are the driving force behind and responsible for the properties and functions of BWO.

The chemical and physical properties of BWO-based materials are closely related to their structures, morphologies and photoluminescence emissions, which are an outcome of the synthetic pathway. Several methods have been developed to synthesize the BWO orthorhombic structure. Previous reports showed that the BWO orthorhombic phase can be obtained by a solid-state reaction^26^, conventional hydrothermal synthesis^8,43^, coprecipitation^44^, sol-gel^45^, the flux growth method^46^, the ultrasonic-assisted method^47^ and the microwave-assisted hydrothermal (MAH) method^48^. Among them, hydrothermal methods are highly chosen because they consume less time and energy without additional heat treatment to obtain pure crystalline phases. Moreover, in the last few decades, it has been observed that the application of microwave irradiation to hydrothermal reactors is a viable alternative for reducing the temperature and time conditions^49^. However, to attain the optimized properties and performance discussed above, knowledge and control over the order/disorder effects and crystallinity on the crystallographic phase are mandatory, which are not fully dominated under microwave hydrothermal conditions.

As an emerging technology, femtosecond (fs) laser irradiation offers a powerful and effective way to not only change material properties and/or structural features but also carry out the preparation and surface modification of materials^50–53^. Periodical structures induced by femtosecond lasers are a unique phenomenon when pulsed lasers irradiate on some material surfaces. These periodical structures with a subwavelength-scale period hold potential in technological applications because of their unique advantages, including multiphonon absorption, a precise ablation threshold, excellent controllability, and a negligible heat-affected zone^54–60^. Fs irradiation allows high-level control of the modification of the morphology and properties in nanofabrication approaches, where the feature size is limited only by the spot size of the laser beam^61–63^. This control is due to the fact that lasers can deposit a large amount of energy per unit area over a temporal scale shorter than the phonon-electron relaxation time^64^. Recently, fs-laser-induced crystallization in glasses^65^, aspirin^66^ and many different kinds of films such as Si^67–70^, Ti^71^, Ge^72^, and diamond^73^, among others^74–79^, has been demonstrated^80^.

A fundamental understanding of the origin and impact of material imperfections, such as structural and electronic order/disorder, has been the key to unlocking their technological potential. To this end, intense efforts to either mitigate and avoid or control and exploit these imperfections have been made. However, synthesis limitations, the presence of impurities, secondary phases, and aggregates, among others, are commonly observed. Therefore, there is still a lack of efficient and easy synthesis to avoid these drawbacks and obtain well-crystallized materials. Very recently, we presented the scale-up of the formation of Ag nanoparticles on α-Ag_2_WO_4_ with bactericidal properties via fs laser irradiation^81,82^ and the formation of Bi and In nanoparticles on NaBiO_3_^83^ and InP^84,85^ under fs irradiation, respectively. This procedure offers the ability to selectively tailor nanomaterials and, in the above cases, to promote properties such as photoluminescence (PL) emissions, with concomitant applications on photodegradation processes and antimicrobial activity. According to our experimental and theoretical results, an intense fs laser pulse excites the electron–lattice system to modify atomic structures and induce the reduction of metal cations non-thermally. Therefore, these unique properties of fs interaction with materials make fs lasers a unique tool for transforming the structures of nanoscale elements without thermally induced structural defects^86–88^. However, to the best of our knowledge, the crystallization and organization of BWO in powder form by fs laser irradiation have not been studied. In this work, we report a remarkable enhancement of the structural organization and crystallinity of BWO, appearing after exposure of the as-synthesized samples by the MAH method to fs laser irradiation.

## Results

### Long-range order

**Figure 1:**
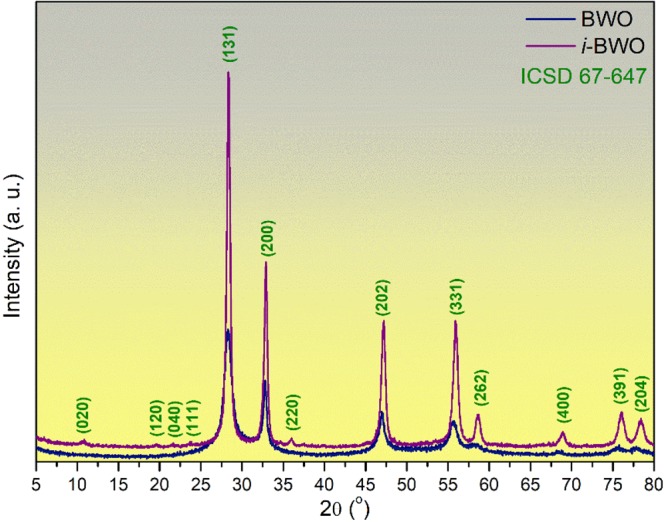
X-ray diffraction patterns of BWO and *i*-BWO crystals.

Figure 1 shows X-ray diffraction (XRD) patterns for the pure Bi_2_WO_6_ (BWO) and fs laser irradiated Bi_2_WO_6_ (*i*-BWO) samples. It is possible to observe sharp diffraction peaks indicating the phase purity and crystallinity of the powder sample. Both compounds presented orthorhombic-type structures with the space group *Pca*2_1_, which agree with the *ICSD n°*67–647 card. Figure 1 clearly shows that there are no additional peaks, even after fs laser irradiation, which indicates that the structure has a single phase. However, the main feature observed is an improvement in the crystallinity of the sample irradiated by the fs laser. An analysis of the full width at half maximum (FWHM) of the most intense peak of the XRD patterns related to the (131) plane was performed to understand the degree of order/disorder of the samples. The BWO and *i*-BWO samples had an FWHM of ~1.24° and 0.53°, respectively. This result indicates an increase in the long-range order for the *i*-BWO sample, which is in agreement with the results of Raman, UV-vis and PL, as will be discussed in the next sections.

Structural properties of the BWO and *i*-BWO samples were assessed by Rietveld refinement method. The refined parameters includes preferred orientation, lattice parameters and shift lattice constants, atomic functional positions among others instrumental and sample parameters. The background was adjusted by a Chebyshev function and the peak profile by a convolution of Thompson-Cox-Hastings pseudo-Voigt (pV-TCH) function. The asymmetry function and the anisotropy in the half-width of the reflections was considered according to Finger *et al*.^89^ and Stephens^90^, respectively.

**Figure 2:**
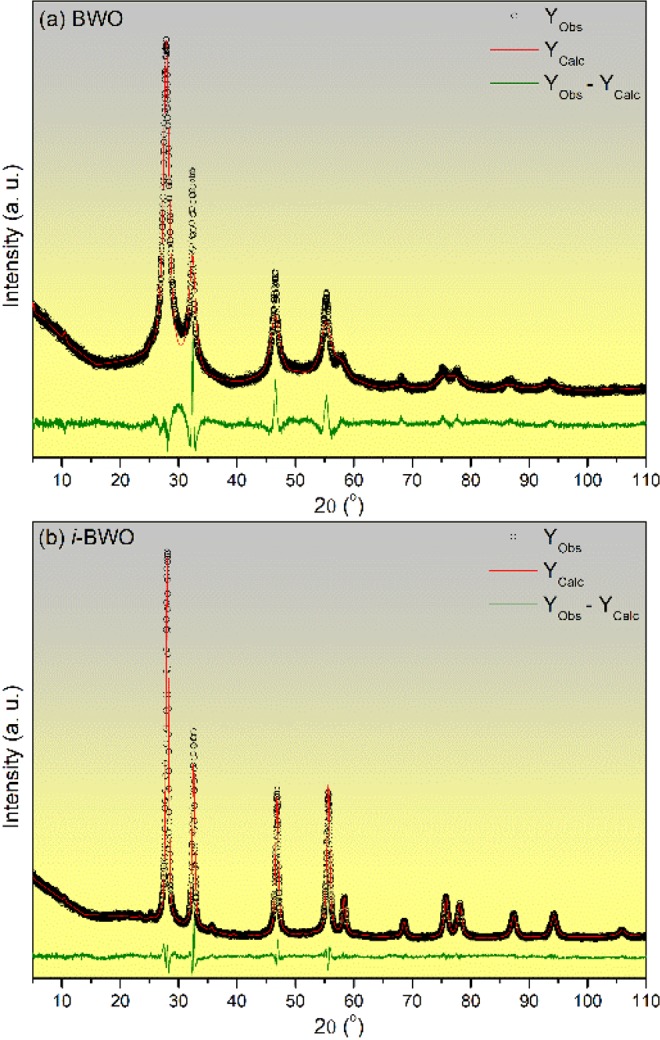
Rietveld refinement plot of (**a**) BWO and (**b**) *i*-BWO.

Figure 2(a,b) shows Rietveld refinement diffractograms of observed *versus* calculated pattern of the samples. The measured diffraction patterns agreed with the *ICSD* card n° 67–647, and the difference between XRD profiles for experimentally observed and theoretically calculated was small, at near zero in the intensity scale, as shown by the line (Y_Obs_ − Y_Calc_). Table 1 list experimental lattice parameters and details on the quality of the structural refinement. It was observed small deviations of the statistical parameters (*R*_wp_ and GOF), attesting that the refinement results are reliable and accurate. Moreover, these data confirm that both samples crystallize in an orthorhombic structure with a symmetry space group termed Hermann-Mauguin (*Pca*2_1_).

**Table 1:** Lattice parameters, unit cell volume, and statistical parameters of quality obtained by Rietveld refinement for Bi_2_WO_6_.

Refined Formula	Lattice Parameters (Å)			V (Å^3^)	R_wp_	GOF
	a	b	c			
BWO	5.500(18)	16.475(0)	5.497(29)	498.0(8)	8.310	2.277
*i*-BWO	5.454(3)	16.392(10)	5.459(5)	488.1(4)	6.398	1.612

A slight decrease in the statistical parameters of the quality values was observed when comparing the BWO sample to the *i*-BWO sample. The fs laser irradiation is the main reason for the differences observed, which promoted a greater interaction between Bi and W polyhedrons. As a consequence, the phase was properly formed, and the amorphous part of these compounds, which causes strain and stress in the orthorhombic structure, was markedly reduced. Moreover, fs laser irradiation dramatically enhances the organization of the [WO_6_] and [Bi_2_O_2_] layers within the crystal lattice, preventing the formation of structural defects (oxygen vacancies, distortion of bonds, stresses, and strains, etc.), which takes place due to the presence of solvent, temperature, pressure, etc., during synthesis. These defects are responsible for the lower crystallinity of the BWO samples with respect to the *i*-BWO samples. The fs laser irradiation acts directly on the electronic distribution around the [BiO_6_] and [WO_6_] clusters as constituent building blocks of BWO, with highly, Bi–O, W–O covalent bonds (long range).

**Figure 3:**
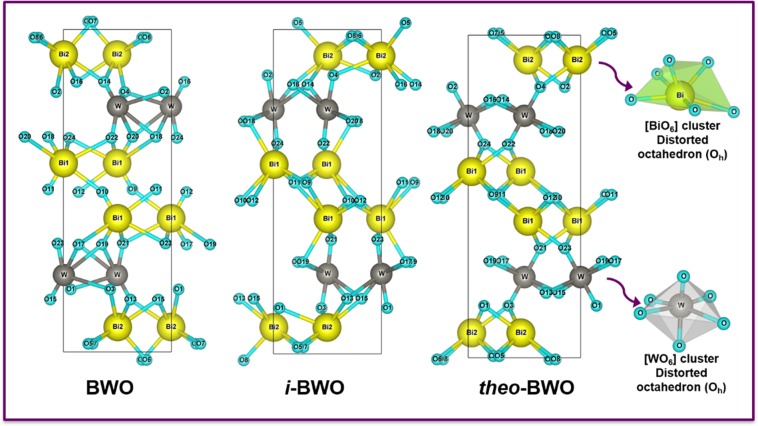
Unit cell representation of Bi_2_WO_6_ for BWO, *i*-BWO, and *theo*-BWO structures. (obtained by VESTA v.3 software - https://jp-minerals.org/vesta/en/).

From these results of the Rietveld refinement for the BWO and *i*-BWO samples, theoretical calculations were performed. The BWO and *i*-BWO calculations were of the single-point type, without any optimization, while the optimized theoretical Bi_2_WO_6_ (*theo*-BWO) structure was optimized, as stated in the theoretical methodology. A representation of the unit cell for these three models with the orthorhombic structure is presented in Fig. 3. These unit cells were modelled using the visualization for electronic and structural analysis (VESTA) program^91,92^, version 3, for the MacOS and were modelled using lattice parameters and atomic positions obtained from the Rietveld refinement data (BWO and *i*-BWO) and from the optimized structure of the theoretical calculation (*theo*-BWO). Figure 3 shows the existence of distorted polyhedrons with octahedral (O_h_) configurations for both the Bi and W sites, which are coordinated to six O atoms. These polyhedrons, namely, the [BiO_6_] and [WO_6_] clusters, correspond to their local coordination and are responsible for the structural and electronic order/disorder effects at the crystal lattice. To verify the order/disorder degrees present in each structure, a full analysis of the distance bonds present in each cluster (Bi−O and W − O) was carried out. These results are presented in Table 2. In addition, more ordered polyhedrons are expected for the *i*-BWO sample, which presented more defined and sharp diffraction peaks, as confirmed by the XRD and Rietveld refinement analyses.

**Table 2:** Bi−O and W − O bond distances (Å) on the [BiO_6_] and [WO_6_] clusters, respectively, for the BWO, *i*-BWO, and *theo*-BWO structures.

Systems		
BWO	*i*-BWO	*theo*-BWO
Bi(1)−O	Bi(1)−O	Bi(1)−O
1.582 1.952 2.476 2.536 2.827 2.868	1.823 2.352 2.416 2.565 2.628 2.768	2.170 2.284 2.299 2.440 2.467 2.728
Bi(2)−O	Bi(2)−O	Bi(2)−O
1.782 2.334 2.406 2.541 2.686	1.882 2.410 2.455 2.466 2.526	2.170 2.284 2.298 2.439 2.731
W−O	W−O	W−O
1.406 1.719 1.788 2.293 2.788 2.943	1.698 1.806 1.822 1.890 2.568 2.751	1.701 1.701 1.824 1.824 2.616 2.618

An analysis of the results of Table 2 and Fig. 3 indicates that the fs laser irradiation induces a larger structural organization in the *i*-BWO system and that a more symmetric structure appears, i.e., the structural order in both the [BiO_6_] and [WO_6_] clusters increases and may lead to lengthening of the Bi–O and W–O bonds, which resembles the perfect system (*theo*-BWO). These theoretical results corroborate the experimental results shown by the defined and sharp diffraction peaks in the XRD and Rietveld refinement analyses. Therefore, the order of symmetry and organization of the systems is *theo*-BWO > *i*-BWO > BWO, as a consequence of the arrangements of the atoms inside the orthorhombic BWO structure.

### Medium-range order

**Figure 4:**
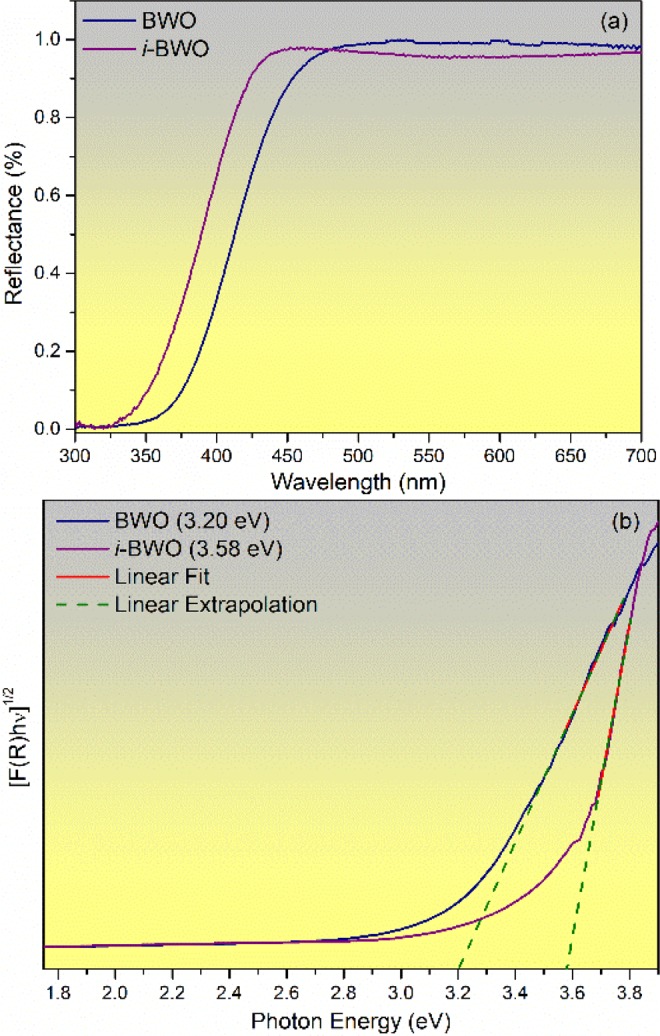
(**a**) UV-vis diffuse reflectance spectra of BWO and *i*-BWO. (**b**) Band gap energy estimated by Kubelka and Munk.

Figure 4(a) illustrates the UV-vis diffuse reflectance spectra of the BWO and *i*-BWO samples within the range of 300–700 nm. The samples showed absorption in the visible range of approximately 375 nm. The absorption is a result of the hybridization of O 2*p* and Bi 6 *s* orbitals forming the valence band (VB), while the conduction band (CB) has major contributions from the W 5*d* orbitals^48^.

**Figure 5:**
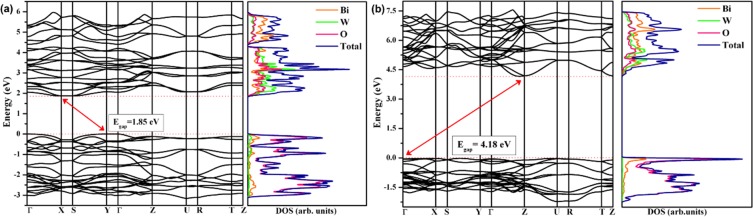
Electronic band structure and density of states for (**a**) *i*-BWO and (**b**) *theo*-BWO models.

The band gap energy (*E*_*gap*_) values were obtained by linear extrapolation of the UV-vis curves in the [F(R_∞_)*hʋ*]^*n*^ *versus hʋ* graph, which were calculated using the relation of the Kubelka-Munk and Wood Tauc function^93,94^. F(R_∞_) is the Kubelka-Munk function, *hʋ* is the photon energy, and *n* is a constant related to the type of electronic transition of a semiconductor, with *n* = 0.5 for direct allowed, *n* = 2 for indirect allowed, *n* = 1.5 for direct forbidden, and *n* = 3 for indirect forbidden. The theoretical calculation predicts an indirect allowed transition for BWO with the *Pca*2_1_ space group, which accounts for n = 2. The *E*_*gap*_ values obtained were 3.20 eV and 3.58 eV for the BWO and *i*-BWO samples, respectively (Fig. 4(b)). The *E*_*gap*_ value for the BWO is in agreement with the values reported in the literature for this orthorhombic structure^48^. The higher band gap energy of the *i*-BWO sample is due to structural organization in the medium range, which favours a large band gap between the VB and CB. These experimental results of *E*_gap_ and the organization of the *i*-BWO in the medium range after fs laser irradiation were confirmed by the theoretical calculations. The theoretical *E*_gap_ values obtained were 0.64 eV, 1.85 eV and 4.18 eV for the BWO, *i*-BWO and *theo*-BWO models, respectively. The huge differences in *E*_gap_ values are due to the structural order/disorder degree for each model, as shown in Table 1. The models present distinguished Bi−O and W − O bond lengths. It is known that the cause of the *E*_gap_ decrease is the creation of new intermediate levels between the VB and CB^95^. Therefore, systems with a higher order of degree present fewer intermediate levels between the VB and CB, which configure the system with a higher band gap value. Figure 5 shows the electronic band structure and the density of states for the *i*-BWO and *theo*-BWO models. The graphics for the BWO model are not illustrated due to difficulty in representing the smaller *E*_gap_ value.

From the analysis of Fig. 5, both the *i*-BWO and *theo*-BWO models present an indirect band gap, where the *i*-BWO model is between the Y and X points and the *theo*-BWO model is between Γ and Z. Despite the fs laser irradiation organizing the *i*-BWO systems, the *theo*-BWO system is still more organized, which is evidenced by the increases in *E*_gap_ obtained for this model. From the density of states analysis, it is possible to note that for both *i*-BWO and *theo*-BWO models, the VB is composed of a major contribution of O atoms, with a minor contribution of Bi and W atoms, and the CB is mainly constituted by W atoms with contributions from the O and Bi atoms.

**Figure 6:**
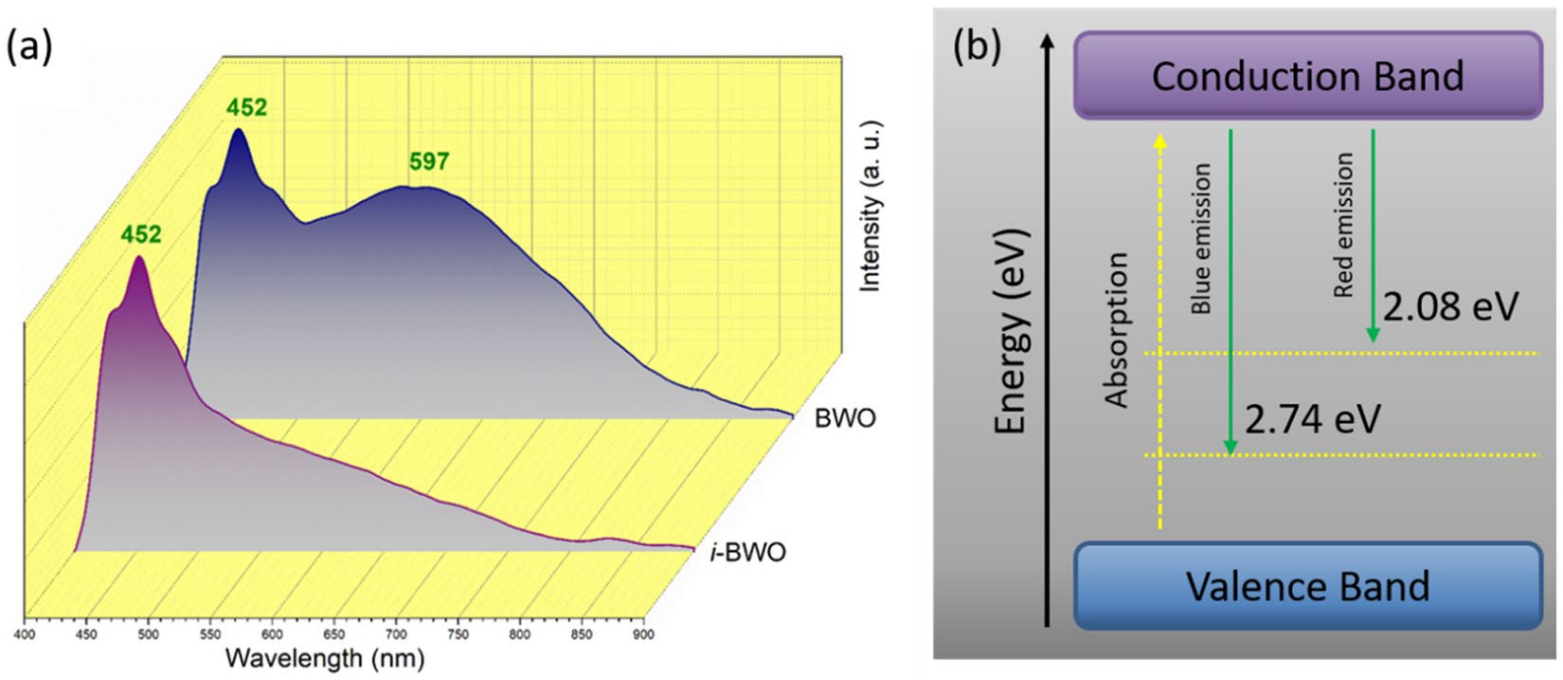
(**a**) Photoluminescence spectra of BWO and *i*-BWO excited at 355 nm. (**b**) Schematic representation of the PL emission related to the presence of the intermediate levels between the conduction band and the valence band. (obtained by OriginPro v.9 software - https://www.originlab.com/Origin).

The PL emission intensity is a result of the presence of defects and different charged transfer processes. Figure 6(a) displays the PL spectra of BWO and *i*-BWO using the excited wavelength of 355 nm. Both samples present a broadband profile covering the entire visible spectrum, centred at 452 nm (2.74 eV) and 597 nm (2.08 eV). This behaviour is due to the radiative transition within the octahedral WO_6_ group, which is characteristic of metal tungstates. Furthermore, this PL profile is typical of a multiphonon process, i.e., a system in which relaxation occurs by various paths, involving the participation of numerous energy levels within the band gap. Based on the molecular orbital theory, the emission bands can be ascribed to the transition from the low vibration level of ^1^T_2_ to the ^1^A_1_ ground state within the WO_6_ excited moiety^96–103^. Figure 6(b) shows a schematic representation of the PL emissions related to the presence of the intermediate levels between the CB and the VB. It is possible to note the disappearance of the band centred at 597 nm after fs laser irradiation. This behaviour can be associated with the presence of oxygen atoms acting as electron acceptors, which avoid the recombination process^104^.

**Figure 7:**
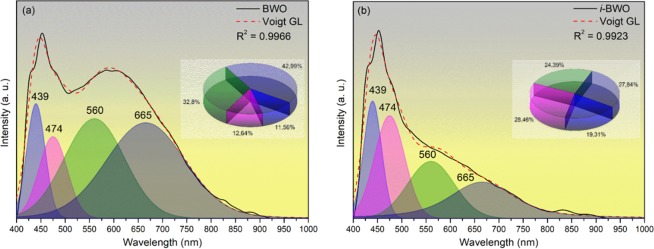
PL deconvolution spectra of (**a**) BWO and (**b**) *i*-BWO. Inset: Percentage of colour area for the components. (obtained by PeakFIT v.4 software - http://www.sigmaplot.co.uk/products/peakfit/peakfit.php).

Figure 7 displays the deconvolution of the PL emissions. The Voigt area G/L function was used for the deconvolution process, resulting in four components centred on the violet (439 nm), blue (474 nm), green (560 nm) and red (665 nm) regions. An analysis of the results indicates that the BWO sample has a larger percentage of emission in the green (32.80%) and red (42.99%) regions, whereas the violet (11.56%) and blue (12.64%) regions present lower percentages. On the other hand, the *i*-BWO sample presents an increased percentage in the violet (19.31%) and blue (28.46%) regions and decreased percentages in the green (24.39%) and red (27.84%) regions compared with the BWO sample. The larger and lower wavelengths of the PL components can be related to the presence of vacancy defects and intrinsic structural defects of the samples, respectively. The higher area components in the green and red regions for the BWO sample indicate the presence of larger numbers of vacancies and defects, respectively, which generate intermediate levels between the VB and CB^105^. Moreover, under fs irradiation, the structure and electronic density of the [BiO_6_] and [WO_6_] clusters and their corresponding networks in the 3D lattice, namely, [WO_6_]-[WO_6_], [BiO_6_]-[BiO_6_] or [WO_6_]-[BiO_6_] complex clusters (medium range), are more symmetric, as confirmed by the data in Table 2. This result is a signature of the enhancement of the structural and electronic organization of the *i*-BWO sample with respect to the BWO sample.

### Short-range order

**Figure 8:**
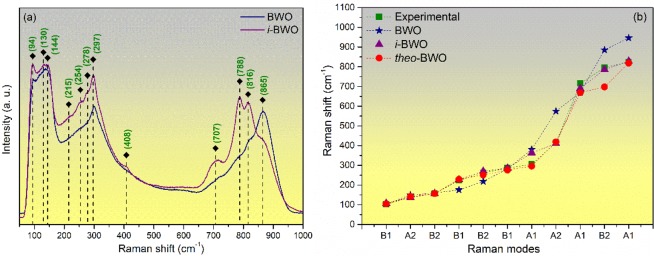
(**a**) Raman spectra of BWO and *i*-BWO excited at 514.5 nm, and (**b**) comparison between relative positions of experimental and theoretical Raman modes for BWO structures.

The structural order in the short range for the samples was determined by Raman spectroscopy. According to factor group analysis, the *Pca2*_1_ structure of Bi_2_WO_6_ presents 105 Raman modes, as stated by the following irreducible representation^106^: Γ = 26A_1_ + 27A_2_ + 26B_1_ + 26B_2_. Figure 8(a) shows the Raman spectra of BWO and *i*-BWO excited at 514.5 nm. Clearly, intense and defined modes are found for the *i*-BWO sample compared with the BWO sample due to the laser irradiation of the former. The modes in the low energy range are centred at 94, 130, 144, 215, 254, 278, 297 and 408 cm^−1^, which can be assigned to bending vibrations of the (Bi_2_O_2_)^2+^ layer, translational motion of the Bi^3+^ cations and vibrations related to the translational motion of Bi^3+^ and W^6+^ cations. Moreover, there are modes with weak intensity in this region, which can be associated with vibrations involving the translational motion of Bi^3+^ and W^6+^ ions. In the region of higher energy, three modes at 707, 788 and 816 cm^−1^ can be observed, which may be assigned to the symmetric and asymmetric stretching vibrations of the (WO_6_) moiety^48,106–110^. The comparison between the relative experimental and theoretical positions of these vibrational modes is illustrated in Fig. 8(b). This figure confirms the good agreement between the experimental and theoretical (*i*-BWO and *theo*-BWO) Raman modes and the structural organization of the *i*-BWO structure.

Thereby, the fs irradiation provokes a structural and electronic organization in the short range in the BWO matrix with concomitant polarization of both [BiO_6_] and [WO_6_] clusters. It is believed that fs laser irradiation enhances the formation of ordered Bi and W clusters which in consequence will present active vibrations as demonstrated in the more defined and intense Raman spectrum for the *i*-BWO sample compared to the BWO sample.

### Morphological analysis

**Figure 9:**
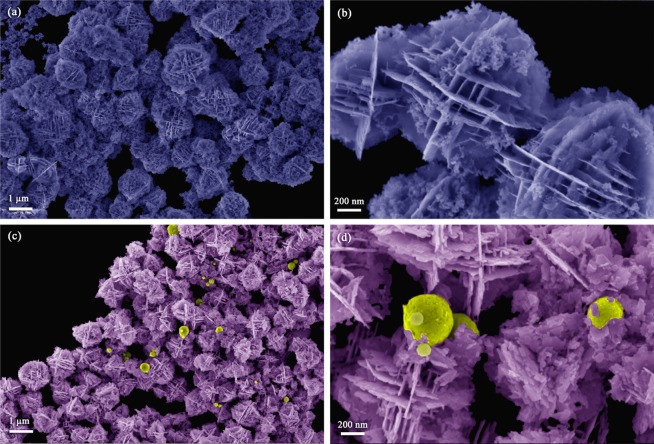
FE-SEM micrographs of (**a**,**b**) BWO and (**c**,**d**) *i*-BWO.

FE-SEM images of BWO and *i*-BWO are shown in Fig. 9(a–d). Both samples present irregular spherical microparticles with a large size dispersion and aggregates. These spheres are similar to flower-like microstructures constructed from nanoplates with a single crystal structure. The formation of these particles proposed by other authors involves three main steps: self-aggregation, Ostwald ripening and self-organization^4,111^. For the *i*-BWO sample shown in Fig. 9(c,d), the formation of small spherical particles with diameters of approximately 500 nm and smooth surfaces was also observed. Similar results were observed in BiEuWO_6_^112^ and copper nanotube^113^ samples under electron beam irradiation. The Bi_2_WO_6_ semiconductor consists of two octahedral clusters with strongly covalent bonds. We analyse the unit-cell structure, local bonding, and band structure of BWO, and fs irradiation induces a decrease in the structural and electronic order as a result of the semiconductor becoming more crystalline^114,115^.

**Figure 10:**
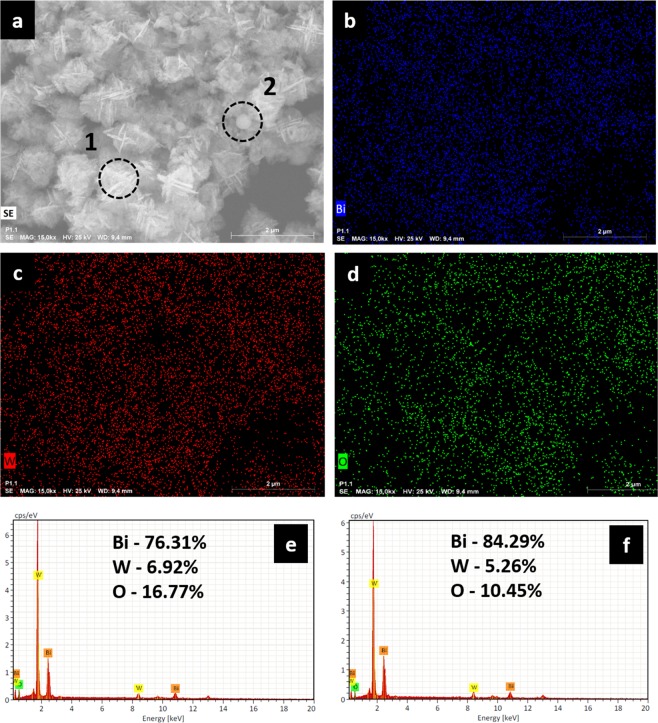
2D mapping and EDS analyses of the *i*-BWO sample. (**a**) FE-SEM micrograph of *i*-BWO, (**b**) Bi points, (**c**) W points, (**d**) O points, (**e**) EDS of region 1 and (**f**) EDS of region 2.

To confirm the elemental composition and distribution uniformity, the elemental 2D maps for the *i*-BWO sample were created and are displayed in Fig. 10(a–d). The presence of Bi, W and O elements over the entire sample is clearly observed. All the elements are homogeneously distributed in the matrix, indicating the purity of the samples.

The Energy dispersive X-ray spectroscopy (EDS) technique was used to check the elemental compositions of the *i*-BWO sample, and the results are shown in Fig. 10(e,f). It can be seen that the *i*-BWO sample is composed of only Bi, W and O elements, and no other element or impurity is found. Quantitative analysis of region 1 (flower-like structures) and region 2 (sphere-like structures) of the *i*-BWO sample is presented in Fig. 10(e,f), respectively. The results are in accordance with the theoretical percentages, and small deviations are due to the region of the analysis, also indicating that both structures are BWO.

**Figure 11:**
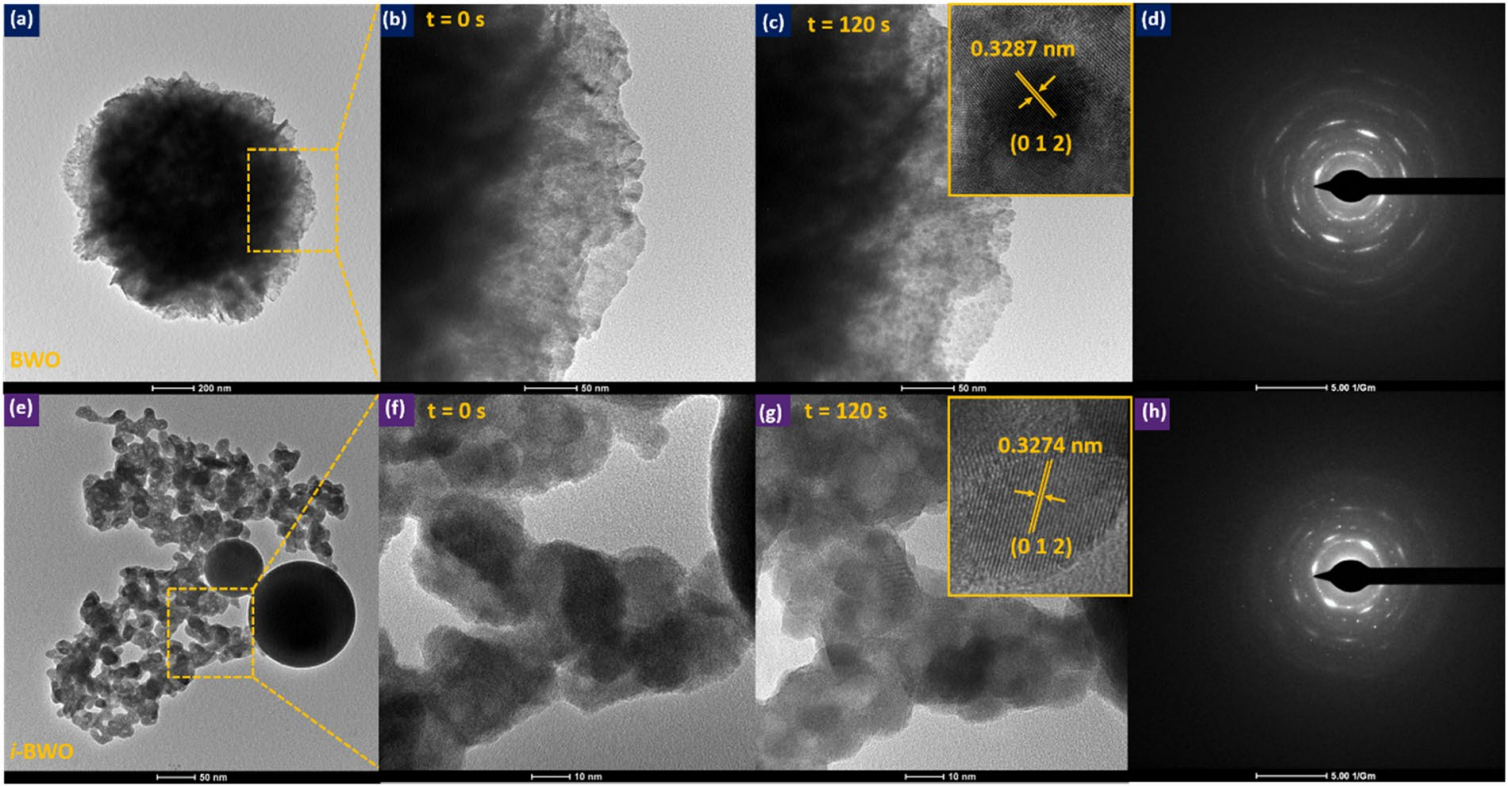
Low magnification TEM image of (**a**) BWO and (**e**) *i*-BWO. (**b**) Electron-beam irradiation at t = 0 s and (**c**) t = 120 s. Inset: HRTEM image of BWO. (**f**) Electron-beam irradiation at t = 0 s and (**g**) t = 120 s. Inset: HRTEM image of *i-*BWO. SAED patterns of (**d**) BWO and (**h**) *i*-BWO.

Different types of crystal defects, including oxygen vacancies and/or bismuth defects, can be generated in the BWO and *i*-BWO samples due to electron beam irradiation. Transmission electron microscopy (TEM) images and the electron irradiation behaviour of both samples are shown in Fig. 11. Figure 11(a,e) show a low-magnification TEM image of BWO and *i*-BWO, respectively. It is possible to observe flower-like aggregate particles with a size of approximately 1186 nm in both samples characterized, whereas spherical particles due to fs laser irradiation are also present in the *i*-BWO sample. Figure 11(b)/(f) and (c)/(g) show the specimen irradiated by a high-energy electron beam (working at 200 kV) at time 0 s and 120 s, respectively. Before irradiation, a smooth surface exhibiting no contrast variation was observed. After irradiation for 120 s, spherical-like structures (ca. 5–9 nm in diameter) with a dark contrast appeared on the surface of the examined irradiated area in both the BWO and *i*-BWO samples. Additionally, these spherical particles tend to aggregate into larger particles in a coalescence process with increasing duration of electron-irradiation exposure^116,117^. The inset of Fig. 11(c) clearly shows these structures, which were identified as metallic Bi and were also observed in other works^27,34,118,119^. The insets of Fig. 11(c,g) illustrate HRTEM images of the BWO and *i*-BWO samples, respectively, where it is possible to see an interplanar distance of 0.3287 nm corresponding to the (012) plane, attesting the rhombohedral Bi space group *R*3*mH*^120^ (*ICSD* n°53–797). Furthermore, apart from the metallic Bi formation as a result of electron-beam irradiation, some of these particles present in the *i*-BWO sample are due to fs laser irradiation, as confirmed by another work^83^. Selected area electron diffraction (SAED) images of BWO and *i*-BWO are presented in Fig. 11(d,h), respectively, where different intensity reflection patterns can be observed, indicating high crystallinity and more than one material, concluded to be the BWO phase and metallic Bi. It is believed that both samples decompose into Bi and WO_3_ particles after the generation of oxygen vacancies due to the electron-beam irradiation process^119^. Adding electrons to the conduction band of BWO, we see that it transforms into a more ordered and crystalline structure, where the pair of Bi–O and W–O bonds become elongated, thus lowering the symmetry of the [BiO_6_] and [WO_6_] clusters.

## Conclusions

It is well known that the structural organization and crystallinity of the BWO orthorhombic structure plays an important role in its performance for practical applications. Here, we report for the first time that symmetry increases in BWO upon application of fs laser irradiation. Long-range order was confirmed by XRD and Rietveld refinement, which showed sharp and well-defined peaks after fs laser irradiation with no segregated materials. UV-vis spectra and PL measurements indicate that the band gap value increases and the PL intensity at larger wavelengths decreases under fs irradiation. Vibrational Raman modes of the *i*-BWO sample are more defined, which represents a signature of the larger structural organization in the short range. The enhancement of the crystallinity after fs irradiation is confirmed by the experimental (X-ray diffraction patterns with Rietveld refinements, UV-vis and Raman spectroscopies, PL emissions), and theoretical (first-principles calculations at the DFT level) results, allowing us to discuss the structural and electronic order/disorder effects of the BWO and *i*-BWO structures and compared with those of the ideal *theo*-BWO structure.

## Experimental Section

### Synthesis

1 mmol of Na_2_WO_4_.2H_2_O (99%, Sigma−Aldrich) was dissolved in 40 mL of distilled water and heated at 50 °C under magnetic stirring until the reagent was dissolved completely. Additionally, 2 mmol of Bi(NO_3_)_3_.5H_2_O (≥98.0%, Sigma-Aldrich) was dissolved in 40 mL of ethylene glycol (≥99.0%, Neon) at room temperature. After complete dissolution of the reactants, the W solution was mixed with the Bi solution at room temperature, and the pH was adjusted to 7 via the dropwise addition of a 2 mol/L NaOH aqueous solution. Subsequently, the mixture was stirred for 10 min, and thereafter, it was transferred to the MAH system at 160 °C for 32 min. The precipitates formed were collected at room temperature, washed with distilled water until the pH was neutralized, and dried in a conventional furnace at 60 °C for 12 h. Later, some of the samples were irradiated by the femtosecond laser. The obtained samples were denoted as BWO and *i*-BWO, corresponding to the pure BWO and fs-irradiated BWO samples, respectively.

### Characterization

All measurements were performed at room temperature. The BWO crystals were structurally characterized by X-ray diffraction (XRD) patterns using a D/Max-2000PC diffractometer Rigaku (Japan) with Cu Ka radiation (λ = 1.5406 Å) in the 2θ range from 5° to 80 ° in the normal routine with a scanning velocity of 2°/min and from 5° to 110 ° in the Rietveld routine with a scanning velocity of 1°/min and was refined using the GSAS-II software^121^. Micro-Raman spectroscopy was conducted on a Horiba Jobin-Yvon (Japan) spectrometer charge-coupled device detector and argon-ion laser (Melles Griot, United States) operating at 514.5 nm with a maximum power of 200 mW. UV-vis spectra were taken using a (Varian, USA) spectrophotometer (model Cary 5 G) in diffuse-reflectance mode. Photoluminescence (PL) measurements were performed at room temperature with the samples excited by a 355 nm laser (Cobolt/Zouk) focused on a 20 µm spot. The backscattered luminescence was dispersed by a 20 cm spectrometer, with the signal detected by a charged coupled device detector (Andor technologies). Deconvolution of the PL spectra was obtained using PeakFIT version 4 and OriginPro version 9.0 software. Morphological analysis of the particles was recorded via field emission scanning electron microscopy (FE-SEM) using a Carl Zeiss microscope (Model Supra 35) operated at an accelerating voltage of 30 kV and a working distance of 3.7 mm. Energy dispersive X-ray spectroscopy (EDS) and 2D mapping were carried out in a Bruker-Philips XL-30 FE-SEM Transmission electron microscopy (TEM) analysis was performed using a Jeol JEM-2100F with a field-emission gun (FEG) operating at 200 kV.

### Fs laser irradiation

The fs laser irradiation of the crystals was performed with a Ti:sapphire laser (Femtopower Compact Pro, Femto Lasers) emitting pulses of 30 fs full width at half maximum (FWHM), with a repetition rate of 1 kHz and a central wavelength of 800 nm. Control over the pulse duration was obtained by means of an acousto-optic programmable filter (DAZZLER, Faslite) that compensates for dispersion effects and ensures a pulse duration of 30 fs at the sample plane. An iris was used to spatially filter the beam to a diameter of 6 mm at 1/e^2^, and then it was focused onto the surface of the powder target by an achromatic 75 mm lens. In every case, the sample was placed at the bottom of a quartz cuvette attached to a 3-dimensional programmable linear stage. The irradiation was performed at a constant velocity of 0.5 mm/s with a mean laser power of 200 mW. The process was repeated three times to ensure homogeneous irradiation of the samples.

### Computational methods and model systems

For the theoretical investigation of the femtosecond laser irradiation effects on the structure and electronic properties of Bi_2_WO_6_, three models were modulated: the BWO, *i*-BWO and *theo*-BWO, corresponding to the pure BWO, fs-laser-irradiated BWO, and optimized theoretical BWO, respectively. The calculations of the BWO and *i*-BWO samples were conducted as a single-point calculation, with any relaxation of the atoms or lattice parameters, to evaluate the real effect of the femtosecond-laser-irradiation-induced structural organization and crystallinity of Bi_2_WO_6_. On the other hand, in the *theo*-BWO model, calculations were performed with full optimization of the structure to obtain the theoretical structure with the minimum amount of energy, i.e., the most stable structure for the BWO material. Therefore, the structural and electronic properties of the models were simulated using the CRYSTAL 17^122,123^ package associated with density functional theory (DFT) with the B3LYP functional hybrid^124,125^. The Bi and W atomic centres were described by the pseudopotential basis sets of Weihrich and Cora, respectively, and the O atoms were described using 6–31d1, which was taken from the CRYSTAL website^126^. The level of accuracy on the convergence criteria for bi-electronic integrals was controlled by a set of five thresholds (10^−8^, 10^−8^, 10^−8^, 10^−8^, and 10^−16^). The shrinking factor was set to 8, corresponding to 125 k-points in the irreducible Brillouin zone. The vibration models at the Γ point were evaluated using the numerical second derivatives of the total energies. The electronic band structure and density of states were analysed with the same k-point sampling employed for the diagonalization of the Fock matrix in the optimization process.
